# RIPK1 protects hepatocytes from death in Fas-induced hepatitis

**DOI:** 10.1038/s41598-017-09789-8

**Published:** 2017-08-23

**Authors:** Aveline Filliol, Muhammad Farooq, Claire Piquet-Pellorce, Valentine Genet, Marie-Thérèse Dimanche-Boitrel, Peter Vandenabeele, Mathieu J. M. Bertrand, Michel Samson, Jacques Le Seyec

**Affiliations:** 1 Institut National de la Santé et de la Recherche Médicale (Inserm), U.1085, Institut de Recherche en Santé, Environnement et Travail (IRSET), F-35043 Rennes, France; 2 Université de Rennes 1, F-35043 Rennes, France; 3 Structure Fédérative BioSit UMS 3480 CNRS-US18 Inserm, F-35043 Rennes, France; 4 Inflammation Research Center, VIB, Technologiepark 927, Zwijnaarde-Ghent, 9052 Belgium; 5 Department of Biomedical Molecular Biology, Ghent University, Technologiepark 927, Zwijnaarde-Ghent, 9052 Belgium

## Abstract

Hepatocyte death is a central event during liver disease progression, in which immune cells play key roles by activating members of the Tumor Necrosis Factor Receptor Superfamily (TNFRSF), including TNFR1 (TNFRSF1A), Fas (TNFRSF6) and TRAIL-R2 (TNFRSF10B). Receptor Interacting Protein Kinase 1 (RIPK1) emerged as a signaling node downstream of these receptors. In the case of TNFR1, RIPK1 has been demonstrated to paradoxically serve as a scaffold to promote the survival of hepatocytes and as a kinase to kill them. To evaluate whether RIPK1 also protects hepatocytes from death in response to FasL or TRAIL, we took advantage of liver parenchymal cell-specific *Ripk1* knockout mice (*Ripk1* ^LPC-KO^). We found that *Ripk1* ^LPC-KO^ mice, as well as primary hepatocytes derived from them, were more susceptible to Fas-mediated apoptosis than their respective WT counterparts. Fas-induced hepatocyte death was independent of TNF-α signaling. Interestingly, while TRAIL administration did not induce hepatitis in *Ripk1* ^LPC-KO^ mice or in their WT counterparts, its combination with IFN-γ only induced TNF-α dependent apoptosis in the *Ripk1* ^LPC-KO^ mice. Together, our data demonstrate the protective role of RIPK1 downstream of Fas and highlight the general protective function of RIPK1 in hepatocytes exposed to inflammatory conditions, where TNF-α, FasL and/or TRAIL are present.

## Introduction

The intravenous injection of the plant lectin Concanavalin A (ConA) is a widely used experimental model for acute immune-mediated hepatitis in mice. The ConA carbohydrate-binding protein is known to induce both activation and recruitment of immune cells (such as natural killer T (NKT) –cells) to the liver where they drive inflammation and death of hepatocytes. Accordingly, depletion of NKT-lymphocytes or of the invariant-NKT subpopulation avoids liver injury normally induced by ConA^1–3^. During liver diseases, members of the Tumor Necrosis Factor Superfamily (TNFSF), such as TNF-α, FasL (or Apo1/CD95) and TNF-Related Apoptosis Inducing Ligand (TRAIL) are expressed and released by immune cells, and can directly or indirectly trigger hepatocyte death^4, 5^. TNF-α and IFN-γ indirectly participate in liver injury by promoting favorable inflammatory conditions dependent on non-parenchymal cells. Indeed, specific deletion of TNFR1 in myeloid-derived cells, but not in liver parenchymal cells, protects from ConA-induced hepatitis^6^, while IFN-γ has been shown to promote NKT-activation^7^. In contrast, TRAIL and FasL, the main cytokines expressed by the hepatic NK- and NKT-cells in the ConA model, are most likely the cytokines directly responsible for the death of hepatocytes since their inhibition or deletion prevent ConA-induced liver damage^2, 8–12^. In line with this idea, the mouse liver is highly sensitive to a single injection of FasL^13^ or of a Fas agonist^14^. It induces fulminant hepatitis by triggering acute hepatocyte and liver sinusoidal endothelial cell (LSEC) death in a dose dependent manner, leading to liver hemorrhages and death of the mice^13–15^. A single injection of TRAIL does not induce hepatolysis, but inhibition of the TRAIL pathway protects mice against ConA-induced liver damage^8, 12^, supporting a pro-death role played by TRAIL in the inflammatory context generated by ConA.

The Receptor Interacting Protein Kinase 1 (RIPK1) has been identified as part of the cellular responses induced by members of the TNF superfamily, including TNF-α^16^, FasL^17^ and TRAIL^18, 19^. Intense efforts have been made in the last decade to better understand the role of RIPK1 in TNF-α signaling, where it was reported to serve as a signaling node controlling the life/death cell-fate switch^20–22^. RIPK1 was shown to function as a scaffold to promote TNF-α-mediated cell survival through canonical NF-κB activation or by a NF-κB independent process mediated by TRAF2 and cIAP1 stabilization^23–28^, and as a kinase to initiate apoptotic or necroptotic signaling^21, 29^. Interestingly, recent studies demonstrated that RIPK1 deficiency in hepatocytes severely sensitized mice to ConA-induced hepatitis due to massive hepatocyte apoptosis^30, 31^. The fact that TNF-α inhibition only partially protected these mice from liver injury suggests that RIPK1 also has pro-survival role(s) in other signaling pathways activated in the ConA model. In this study, we evaluated the potential pro-survival scaffolding function of RIPK1 in hepatocytes during FasL or TRAIL signaling.

## Results and Discussion

**Figure 1:**
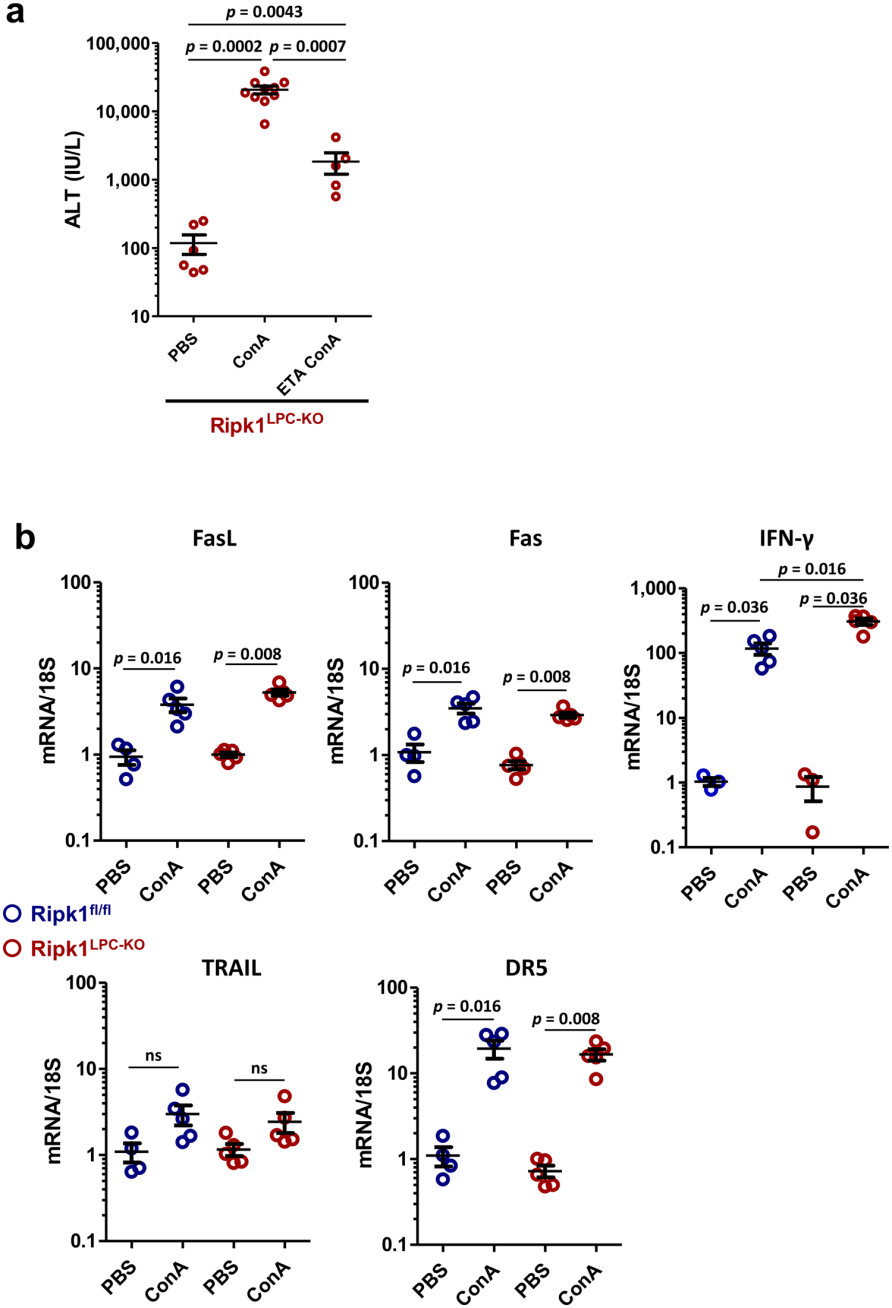
TNF-α inhibition partially protects *Ripk1* ^*LPC-KO*^ mice from ConA-induced liver injuries. (**a**) Levels of serum ALT, 11 h after PBS or ConA injection in *Ripk1* ^*LPC-KO*^ mice, potentially pre-treated with ETA. Number of mice for each group: PBS (n = 6), ConA (n = 10) and ETA + ConA (n = 5). (**b**) Levels of hepatic FasL, Fas, IFN-γ, TRAIL and DR5 transcripts in *Ripk1* ^*fl/fl*^ or *Ripk1* ^*LPC-KO*^ mice 7 h after treatments with PBS or ConA (n = 3–5), (ns: non-significant). For all graphs, each circle represents an individual.

**Figure 2:**
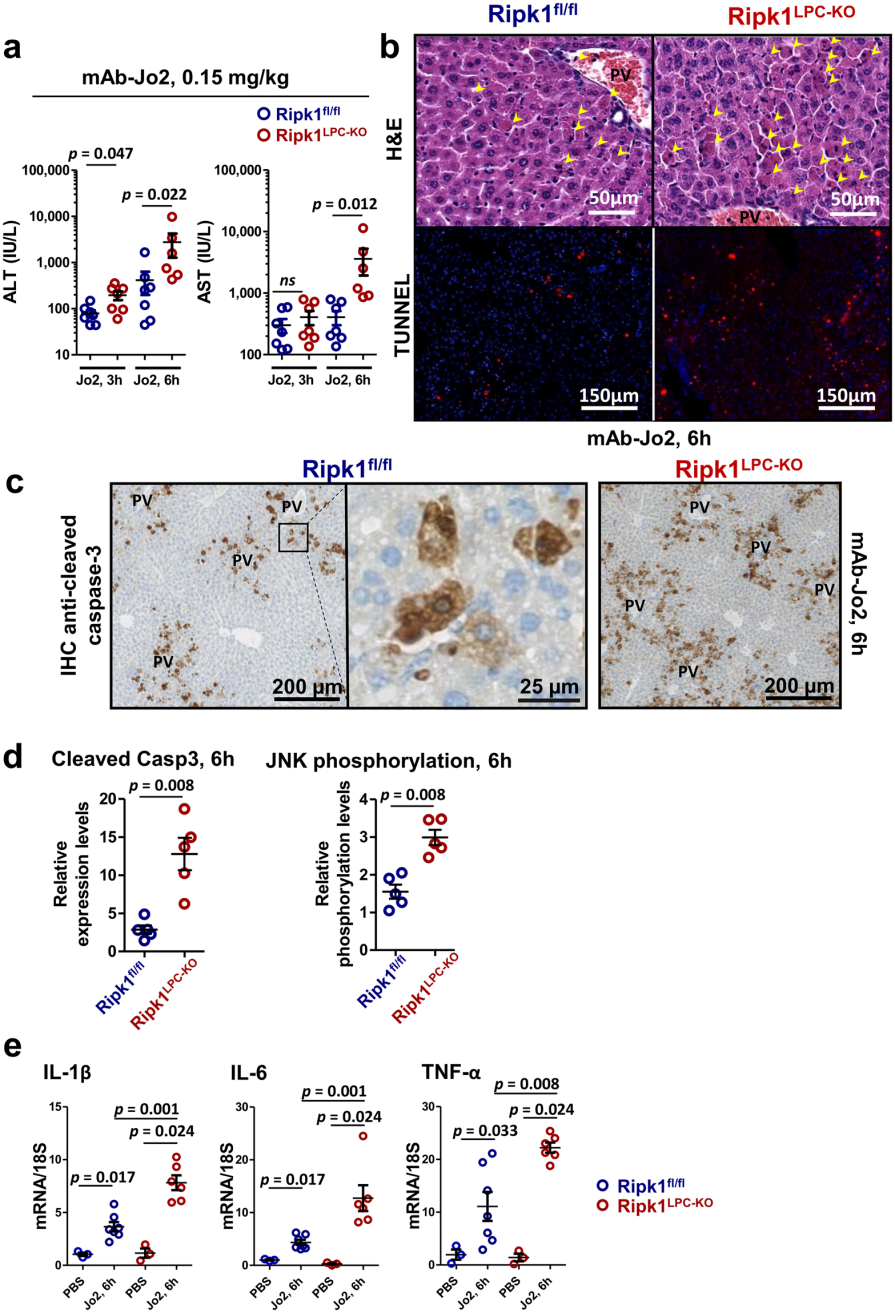
RIPK1 deficiency sensitizes mice to Fas-mediated liver injuries. (**a**) Levels of serum ALT and AST, 3 and 6 h after mAb-Jo2 injection in *Ripk1* ^*fl/fl*^ and *Ripk1* ^*LPC-KO*^ mice (n = 6–7). (**b**) Pictures of liver tissue sections, stained by H&E (upper panels) or analysed by TUNEL (in red) and DAPI (in blue) immunofluorescence (lower panels) issued from *Ripk1* ^*fl/fl*^ and *Ripk1* ^*LPC-KO*^ mice, 6 h after mAb-Jo2 injection. Yellow arrows show necrotic areas, PV: portal vein. (**c**) Immunostaining of cleaved caspase-3 in the livers of *Ripk1* ^*fl/fl*^ and *Ripk1* ^*LPC-KO*^ mice, 6 h after mAb-Jo2 injection. (**d**) Mean levels of cleaved caspase-3 (left panel) and of JNK phosphorylation status (right panel) in the livers of *Ripk1* ^*fl/fl*^ (n = 5) and *Ripk1* ^*LPC-KO*^ (n = 5) mice, collected 6 h after mAb-Jo2 injection (see corresponding Western blots in Supplementary Fig. S1). (**e**) Levels of hepatic IL-1β, IL-6 and TNF-α transcripts in *Ripk1* ^*fl/fl*^ or *Ripk1* ^*LPC-KO*^ mice, 6 h after PBS (n = 3 mice) or mAb-Jo2 injection (n = 6–7 mice). For all graphs, each circle represents an individual.

In previous works, we have described that RIPK1 deficiency in liver parenchymal cells sensitizes mice (*Ripk1* ^*LPC-KO*^) to TNF-α- and by consequence to ConA-induced liver injury^30, 32^. While *Ripk1* ^*LPC-KO*^ mice challenged with ConA developed severe acute hepatitis that sometimes led to death (4 out of the 12 enrolled animals), a co-treatment with Etanercept (ETA), a TNF-α decoy receptor, significantly reduced liver injury and prevented any death. However, ETA did not fully protect mice from hepatitis (Fig. 1a). This suggested that other factors must take part in the hepatocyte death process, potentially such as FasL and/or TRAIL which have been shown to be involved during ConA-induced liver injury in WT mice^2, 8–12^. Thus, the severity of ConA-induced hepatitis could be significantly reduced in presence of FasL antagonists^33^. In agreement with this hypothesis, FasL, Fas and DR5 transcripts were significantly up-regulated in *Ripk1* ^*LPC-KO*^ mice after ConA treatment, as in their WT littermates (*Ripk1* ^*fl/fl*^). Although not significant, TRAIL transcripts also seemed to be slightly upregulated. Besides, levels of mRNA coding for IFN-γ, an important cytokine in this hepatitis model^11^, was more up-regulated in *Ripk1* ^*LPC-KO*^ mice (Fig. 1b). As Fas activation with the Fas-agonist mAb-Jo2 has been shown to induce acute liver injury^14^, we used this hepatitis model to explore the role of RIPK1 under Fas signaling. While injection of a low dose (0,15 mg/kg) of mAb-Jo2 provoked a mild hepatitis in WT mice, the same treatment elicited more important liver damages in *Ripk1* ^*LPC-KO*^ mice, as shown by higher levels of serum transaminases (Fig. 2a) and by the increased number of necrotic areas and of TUNEL positive cells, 6 h after the injection (Fig. 2b). Nevertheless, no individual died from this treatment. In the Fas agonist-induced liver injury model, hepatocytes are believed to die by apoptosis since hepatocyte-specific caspase-8 deficient mice are protected from liver injury^34^. Activation of c-Jun N-terminal kinases (JNKs) is also believed to take part in the apoptotic death process of hepatocytes in different murine hepatitis models^35^, including those induced by Fas^36^. Therefore, we decided to investigate the apoptotic response by assessing the amount of cleaved caspase-3 and by analyzing JNK1/2 activation in the livers of *Ripk1* ^*fl/fl*^ and *Ripk1* ^*LPC-KO*^ mice. As expected, the cleaved caspase-3 labelling revealed positive hepatocytes around the portal veins of WT mice (Fig. 2c). Moreover, activation of JNK1/2, revealed by enhanced phosphorylation, was detected 6 h after mAb-Jo2 administration. Remarkably, both markers were greatly enhanced in the liver of the *Ripk1* ^*LPC-KO*^ mice (Fig. 2d and see Supplementary Fig. S1). Altogether, these data revealed that RIPK1 deficiency in liver parenchymal cells sensitized mice to Fas agonist-induced liver injury due to increased hepatocyte apoptosis. The enhanced hepatocyte death observed in *Ripk1* ^*LPC-KO*^ mice was also associated with an exacerbated liver inflammation, as shown by the increased upregulation of TNF-α, IL-1β and IL-6 transcripts detected in livers after mAb-Jo2 administration, when compared to similarly treated *Ripk1* ^*fl/*fl^ mice (Fig. 2e).

**Figure 3:**
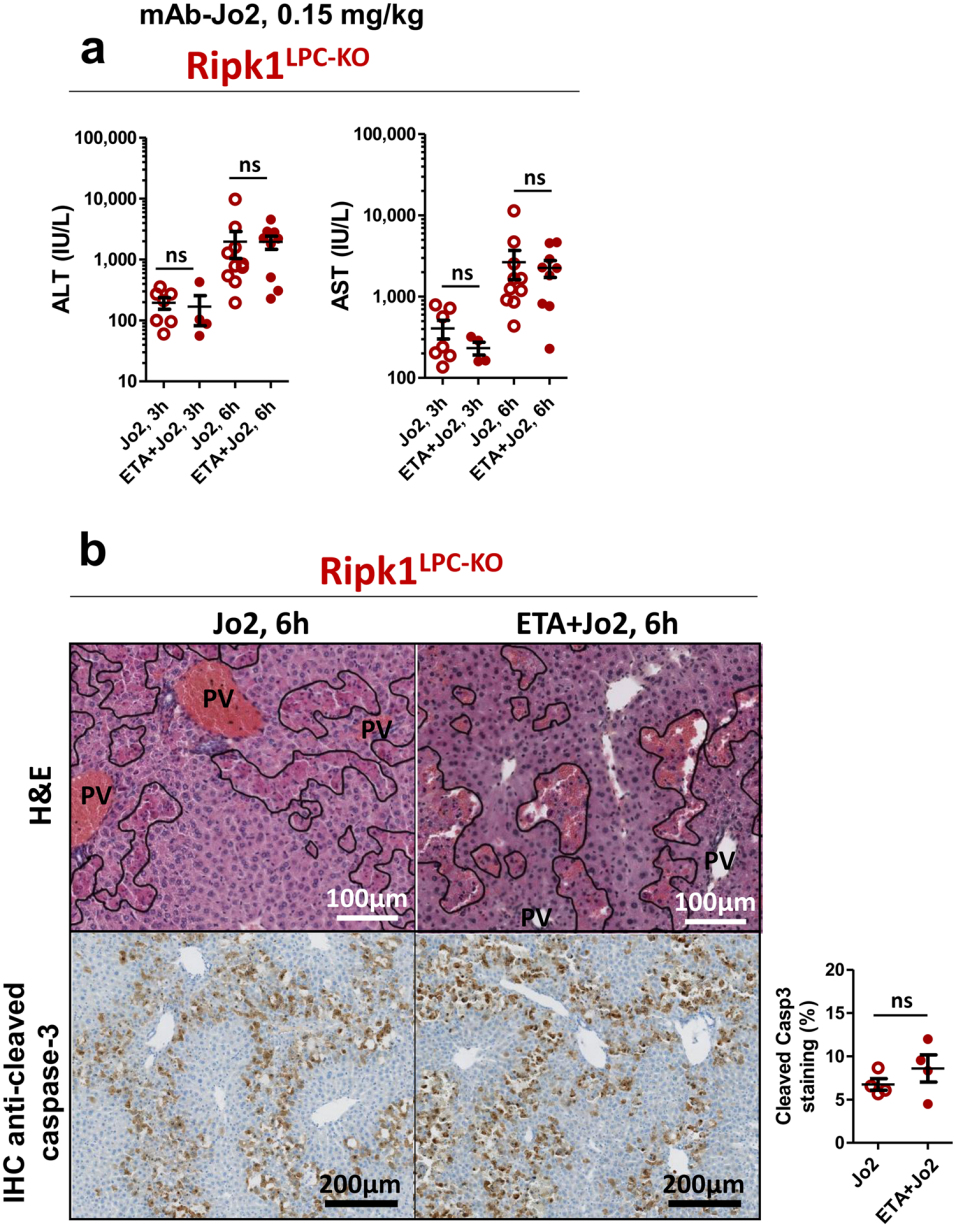
Fas-mediated liver injury in *Ripk1* ^*LPC-KO*^ mice is independent of TNF-α. (**a**) Levels of serum ALT and AST (n = 4–10) in Ripk1^LPC-KO^ mice after 3 or 6 h mAb-Jo2 injection with a possible pre-treatment with ETA (ns: non-significant). Each circle or dot represent an individual. (**b**) Pictures of liver tissue sections, stained by H&E (upper panels) or analysed by IHC for cleaved caspase-3 (lower panels), issued from Ripk1^LPC-KO^ mice, 6 h after mAb-Jo2 injection with a possible pre-treatment with ETA. Signal quantification of cleaved caspase-3 (lower right panel).

Since we previously reported that, unlike their WT counterparts, RIPK1-deficient hepatocytes succumb by apoptosis upon TNF-α sensing, we next investigated the potential contribution of TNF-α in the Fas-agonist induced hepatitis model. Especially since the TNF-α transcript level was even higher in the liver of *Ripk1* ^LPC-KO^ mice (Fig. 2e). In parallel, we found that TNF-α neutralization by prior ETA injection never improved liver injury induced by the Fas-agonist in *Ripk1* ^*LPC-KO*^ mice, as assessed by serum transaminase releasing (Fig. 3a). Accordingly, liver from *Ripk1* ^*LPC-KO*^ mice which received ETA and the mAb-Jo2 displayed large necrotic areas, and a large number of cleaved caspase-3 positive cells, similar to those revealed for *Ripk1* ^*LPC-KO*^ mouse livers only receiving the Fas-agonist (Fig. 3b). All these data demonstrate that the susceptibility of *Ripk1* ^*LPC-KO*^ mice to Fas-induced liver injury is TNF-α independent and thus strongly suggest that RIPK1 has a protective role downstream of Fas. These results contrast with those recently published by Suda *et al*. showing that the *in vivo* Ripk1 knockdown did not affect Fas-induced liver injury^31^. This discrepancy may be explained by the different approaches used. Silencing RIPK1 using antisense oligonucleotides reduces but does not completely abolish the expression of RIPK1. The remaining RIPK1 expression might therefore be sufficient to protect hepatocyte from the liver injury induced by Fas agonist. Moreover, in contrast to our model, Ripk1 knockdown affected not only liver parenchymal cells but also most probably the immune hepatic microenvironment triggered by mAb-Jo2 injection. Finally, some experimental parameters, such as inadequate timing for hepatitis assessment or a too high mAb-Jo2 dose, could also potentially participate in their failure to distinguish the existing difference in Fas-agonist susceptibility between hepatocytes expressing normal or decreased levels of RIPK1.

**Figure 4:**
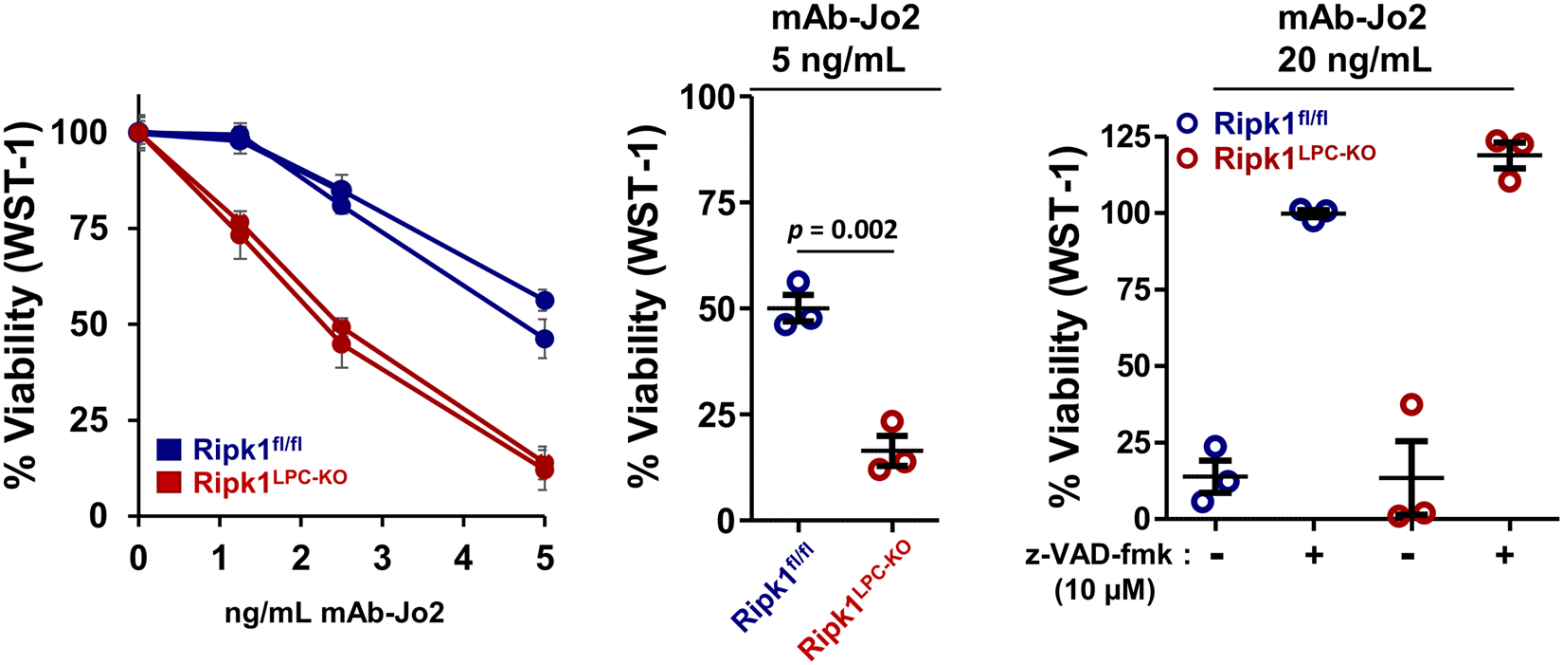
RIPK1 deficiency increases the sensitivity of primary hepatocytes to Fas-agonist stimulation. Primary cultures of hepatocytes issued from *Ripk1* ^*fl/fl*^ (*n* = 2) *or Ripk1* ^*LPC-KO*^ (n = 2) mice were subjected during 16 h to mAb-Jo2 concentrations ranging from 1 to 5 ng/mL in presence of ETA (1 µg/mL) (left panel). Additional primary cultures (n = 3 for each strains) were exposed to a unique dose of mAb-Jo2 (5 ng/mL) also in presence of ETA (1 µg/mL) for statistical analysis (middle panel). In parallel, these primary hepatocyte cultures were subjected during 16 h to mAb-Jo2 at 20 ng/mL in presence of ETA (1 µg/mL) and in absence or presence of the z-VAD-fmk pan-caspase inhibitor (right panel). Cell death was analysed by WST-1 based assay and data are expressed as a percentage of signal obtained in basal survival conditions without Fas-agonist. Error bars corresponds either to internal triplicates for each primary cultures (left panel) or to triplicates of independent primary cultures (middle and right panels).

To further evaluate the protective role of RIPK1 downstream of Fas, we analysed the response of primary cultures of mouse hepatocytes derived from *Ripk1* ^*LPC-KO*^ and *Ripk1* ^*fl/fl*^ mice to Fas activation. The primary hepatocyte cultures were subjected to increasing concentrations of mAb-Jo2 in presence of ETA to avoid any risk of death of RIPK1-deficient hepatocytes originating from TNF-α sensing, as previously reported^30–32^ and shown in Supplementary Fig. S2. Hepatocytes issued either from *Ripk1* ^*fl/fl*^ or *Ripk1* ^*LPC-KO*^ mice died after Fas activation. Nevertheless, cell death was systematically enhanced in *Ripk1* ^*LPC-KO*^ hepatocytes (Fig. 4, left panel). A statistical analysis performed on the dose of 5 ng/mL demonstrated the significant increased sensitivity of RIPK1-deficient hepatocytes to Fas agonist-induced death (Fig. 4, middle panel). In both genotypes, cells died by apoptosis since pretreatment with the pan-caspase inhibitor z-VAD-fmk (10 µM) completely protected both cell populations from death induced by mAb-Jo2 at 5 or 20 ng/mL (Fig. 4, right panel and data not shown). Together our *in vitro* and *in vivo* results demonstrated the protective role played by RIPK1 downstream of Fas in hepatocytes.

**Figure 5:**
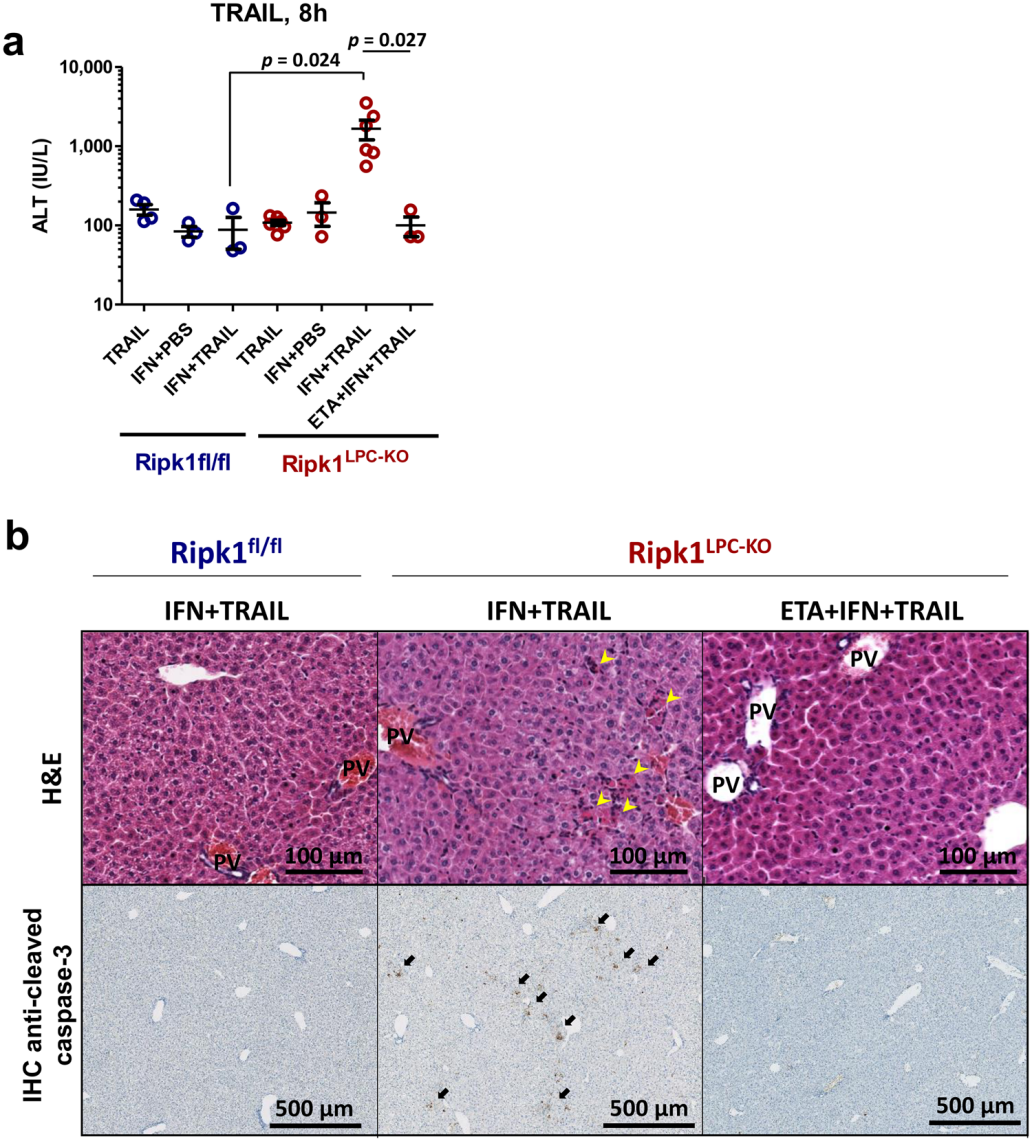
Co-administration of IFN-γ and TRAIL promotes liver injury in *Ripk1* ^*LPC-KO*^ mice in a TNF-α dependent manner. (**a**) Levels of serum ALT, 8 h after TRAIL or PBS injection with a possible pre-treatment with IFN-γ and/or ETA in *Ripk1* ^*fl/fl*^ and *Ripk1* ^*LPC-KO*^ mice (n = 3–6 mice). Each circle represents an individual. (**b**) Pictures of liver tissue sections, stained by H&E (upper panels) or analysed by IHC for cleaved caspase-3 (lower panels), issued from *Ripk1* ^*fl/fl*^ and *Ripk1* ^LPC-KO^ mice, 8 h after TRAIL injection, pre-treated 2 h before with IFN-γ and with a possible pre-treatment with ETA. Yellow and black arrows respectively show necrotic areas and apoptotic hepatocytes, PV: portal vein.

During ConA hepatitis, TRAIL has also been shown to contribute in the liver injury process^8, 12^, but implied mechanisms are not fully understood. Indeed, unlike Fas-agonist, *in vivo* treatment with recombinant TRAIL never induces hepatolysis^37^. Zheng *et al*. have proposed that the microenvironment induced by ConA sensitizes hepatocytes to TRAIL-induced cell death^12^. As IFN-γ has been shown to be implied during ConA hepatitis^11, 38, 39^ and since this cytokine also sensitized some cells to TRAIL-induced apoptosis^40, 41^, we decided to investigate the *in vivo* role of RIPK1 under TRAIL and IFN-γ stimulation. Whereas single injections of TRAIL or IFN-γ did not induce hepatolysis in *Ripk1* ^*fl/fl*^ or in *Ripk1* ^*LPC-KO*^ mice, their co-injection was able to trigger hepatocyte apoptosis but only in *Ripk1* ^*LPC-KO*^ mice, as assessed by the levels of serum transaminases, and appearance of necrotic and cleaved caspase-3 positive cells in the liver of these mice (Fig. 5). During this experiment, no individual died whatever the treatment combinations. Despite relatively high levels of ALT in the sera of *Ripk1* ^*LPC-KO*^ mice co-treated by TRAIL and IFN-γ (around 2,000 IU/L), their livers did not exhibit many cleaved caspase-3 positive cells. Further investigations will be required to determine, for example, whether cell death other than apoptotis occurred. Interestingly, and in contrast to Fas-agonist administration, the hepatolysis induced by TRAIL + IFN-γ was completely prevented by the use of ETA. These results therefore strongly suggest that co-stimulation with IFN-γ and TRAIL triggered TNF-α release that in turn induced hepatocyte apoptosis caused by the lack of RIPK1, as previously reported^30, 31^. Accordingly, low levels of serum TNF-α were detected 8 h after TRAIL injection in *Ripk1* ^*LPC-KO*^ mice pre-treated with IFN-γ (9.23 ± 1.08 pg/mL).

Finally, our data showed that in hepatocytes RIPK1 is able to limit the hepatic injuries initiated during Fas pathway activation which is independent of TNF-α. Consequently, this suggested that the remaining hepatolysis observed in *Ripk1* ^*LPC-KO*^ mice treated with ETA and ConA (Fig. 1a) was most likely dependent on FasL. Likewise to TNF-α, FasL is known to be involved in several liver diseases. As a consequence, our data consolidate the protective role of RIPK1 in dysimmune hepatitis and highlight the risks of potential defects in the RIPK1 scaffolding function that would sensitize hepatocytes to death, risking to worsen hepatitis and even to increase HCC onset.

## Methods

### Animals and treatment protocols

All experimental protocols on animals were conducted in compliance with French laws and the institution’s guidelines for animal welfare (authors were authorized to conduct animal experimentation by “La direction des Services Vétérinaires” (license M Samson #A3523840), the project was authorized by the “Comité Régional d’Ethique d’Expérimentation Animal CREAA, license given by the “Ministère de l'éducation Nationale et de la Recherche” #7576). *Ripk1* ^LPC-KO^ mice have been already described in previous works^30^. In all experiments, genetically modified mice were systematically compared to their WT *Ripk1* ^fl/fl^ littermates. Homogeneous groups of male and female mice at 7 to 13 weeks of age were used for each experiment. ConA (Sigma-Aldrich, C2010) diluted at 3 mg/mL in PBS supplemented with MnCl_2_ 0.31 mM and CaCl_2_ 0.75 mM, was administered by intravenous (i.v.) injection at a dose of 12 mg/kg body weight. Mice fasted for 24 h received Fas agonist (mAb, Jo2) antibody (BD Pharmingen, #554255) via intraperitoneal (i.p.) route at a dose of 0.15 mg/kg body weight (10 µL/g body weight). Recombinant-murine TRAIL (rm-TRAIL) (Peprotech, 315-19) was injected by i.v. route at a dose of 1.5 mg/kg body weight (4 µL/g body weight), 2 h after i.v. injection of IFN-γ (Peprotech, AF-315-05) at a dose of 0.35 mg/kg body weight or of PBS, as a control. An i.p. injection was used to deliver ETA (Enbrel, Pfizer) to mice at a dose of 10 mg/kg body weight (10 µL/g body weight), 1 h before mAb-Jo2 or recombinant murine IFN-γ injection. Mice were sacrificed at the indicated time.

### Histopathological and biochemical studies

Fragments of mouse livers were fixed in 4% paraformaldehyde and embedded in paraffin for IHC and hematoxylin and eosin (H&E) staining. For histopathology, H&E staining of liver tissues was carried out to investigate liver injury. Serum aspartate and alanine transaminases (ALT and AST, respectively) were measured according to the IFCC primary reference procedures using Olympus AU2700 Autoanalyser^®^ (Olympus Optical, Tokyo, Japan).

### Immunolocalization in liver tissues

For immunolocalization of cleaved caspase-3 in liver tissues, paraffin-embedded mouse liver sections (5 µm) were dried 1 h at 58 °C, followed by antigen retrieval and incubated with primary antibody (Cell Signaling, 9661S) in a Ventana automated staining platform (Ventana Medical Systems, USA). Revelation of primary antibody was carried out using horseradish peroxidase (HRP)-conjugated secondary antibody (Dako, USA) and DAB substrate kit (Ventana, #760-124). Slides were then counterstained with hematoxylin. TUNEL analysis was performed on paraffin-embedded mouse liver sections (5 µm), incubated after antigen retrieval with a mix, composed of terminal transferase (Roche, #3333566011) and digoxigenin-11-UTP (Roche, #1558706) followed by HRP-anti-digoxigenin (Ventana, #760-4822). Revelation was used according to the manufacturer’s instructions with the Discovery Rhodamine kit (Ventana #760-233) followed by nucleus labelling with DAPI.

All paraffin-embedded mouse liver sections were scanned with a digital slide scanner (Hamamatsu, Nanozoomer 2.0-RS) and files were analysed with the NDP viewer software. Quantification of cleaved caspase-3 positive-signal was performed with an image analysis software (NIS-Element AR analysis software, Nikon, Tokyo, Japan) and measured to cover an area of 3.9–5.7 mm².

### RNA analysis

Fragments of mouse livers were collected after sacrifices and frozen in liquid nitrogen, and conserved at −80 °C. Total RNA was extracted from mice livers using TRIzol reagent (Invitrogen). First-strand cDNA was synthesized using the SuperScript^TM^ II Reverse Transcriptase (Invitrogen). Real-time quantitative PCR was performed using the fluorescent SYBR Green dye (Applied Biosystems) and the ABI 7000 Prism sequence detector (Applied Biosystems) or the CFX384 Touch™ Real-Time PCR Detection System (Bio-Rad). cDNA was used as a template for amplification with specific primer pairs (Table 1). All measurements were performed in triplicate. The relative gene expression was normalized against the 18S gene expression. The control mice in each treatment group served as a reference for messenger RNA (mRNA) expression (control mRNA level was arbitrarily set at 1).

**Table 1:** Primer sequences used for qPCR.

Gene	Forward	Reverse
Mouse 18S	5′-CGCCGCTAGAGGTGAAATTC-3′	5′-TTGGCAAATGCTTTCGCTC-3′
Mouse TNFα	5′-TAGCTCCCAGAAAAGCAAGC-3′	5′-TTTTCTGGAGGGAGATGTGG-3′
Mouse IL-6	5′-CCGGAGAGGAGACTTCACAG-3′	5′-CAGAATTGCCATTGCACAAC-3′
Mouse IL-1β	5′-GAAGAAGTGCCCATCCTCTG-3′	5′-AGCTCATATGGGTCCGACAG-3′
Mouse IFNγ	5′-AGGTCAACAACCCACAGGTC-3′	5′-ATCAGCAGCGACTCCTTTTC-3′
Mouse FasL	5′-GCAGCAGCCCATGAATTACC-3′	5′-AGATGAAGTGGCACTGCTGTCTAC-3′
Mouse Fas	5′-CTCCGAGTTTAAAGCTGAGG-3′	5′-TGTACTCCTTCCCTTCTGTGC-3′
Mouse TRAIL	5′-CCCTGCTTGCAGGTTAAGAG-3′	5′-GGCCTAAGGTCTTTCCATCC-3′
Mouse DR5	5′-TGACGGGGAAGAGGAACTGA-3′	5′-GGCTTTGACCATTTGGATCT-3′

### Protein extraction and western blotting

Mouse liver specimens were lysed in RIPA buffer (50 mM Tris-HCl pH 7.4; 1% Triton X-100; 25 mM HEPES; 150 mM NaCl; 0,2% SDS; 5 mM MgCl_2_; 1 mM Na_3_VO_4_; 1 mM NaF) containing protease inhibitors (Roche, #04 693 132 001) using an Ultra-Turrax® homogenizer. After 40 min in ice, samples were centrifuged at 15,000 g. Proteins from diluted supernatant were assayed with the Bradford method (BioRad). Proteins were separated by SDS-PAGE and transferred onto nitrocellulose membrane. Membranes were blocked with non-fat milk in TBS (20 mM Tris, 137 mM NaCl) during 1–2 h and incubated overnight at 4 °C with anti-cleaved caspase-3, anti-RIPK1 (Cell Signaling, 3493) anti-actin (Sigma A3854), anti-phospho-Thr183/Tyr185-JNK (Cell Signaling, 9251) or anti-JNK (Calbiochem, 559304) primary antibodies, and then with secondary goat anti-rabbit immunoglobulins/HRP (Dako, P0448). Protein-antibody complexes were revealed by enhanced chemiluminescence (Millipore) and ImageQuant LAS-4000 mini imager analysis (GE-Healthcare). The Multi Gauge software was used for signal quantifications and data was expressed as levels relative to signals detected in PBS controls. Cleaved caspase-3 expression levels or JNK phosphorylation status were respectively normalized on β-actin expression levels or on total JNK expression levels.

### Serum cytokine dosage

Murine TNF-α was quantified by ELISA (Peprotech, 900-M54) or bead-based immunoassays according to manufacturer protocol, using a filter plate and a vacuum filtration system for washing steps (BioLegend’s LEGENDPLEX, multi-analyte flow assay kit). Samples were analysed on a LSR Fortessa cytometer (BD Biosciences).

### Primary hepatocyte isolation and culture

Murine hepatocytes were isolated and purified from adult *Ripk1* ^*fl/fl*^ or *Ripk1* ^*LPC-KO*^ mice as previously described^30^ with some minor modifications. The perfused liver was first washed with solution I (8 g/l NaCl, 0.2 g/l KCl, 0.1 g/l Na_2_HPO_4_. 12 H_2_O, 2.38 g/l Hepes, pH 7.6 and 0.5 mM EGTA) at a 10 mL/min flow rate for 8–10 min. Then, the perfusion solution I without EGTA was supplemented with 5 mM CaCl_2_.2H_2_O and 0.01% collagenase, type 4 (Worthington Biochemical Corporation, Serlabo Technologies, Entraigues, France) at a 7 mL/min flow rate for 5–7 min. After the completed isolation process, hepatocytes were seeded at a density of 6 × 10^4^ cells/cm^2^ in 96-well plates, previously coated with collagen type I (BD Biosciences, Le Pont de Claix, France), in Williams’ E medium supplemented with 10% (vol/vol) fetal calf serum, 2 mM glutamine, 10 IU/mL penicillin, 10 μg/mL streptomycin, 5 μg/mL insulin and 1 μg/mL of ETA. Around 4 h post-plating, cells were washed 2 times with PBS before their stimulation with TNF-α or mAb-Jo2 in a similar medium to the seeding supplemented Williams’ E medium in which fetal calf serum was replaced by 1 mg/mL bovine serum albumin and also potentially containing 1 μg/mL of ETA and/or 10 µM of z-VAD-fmk (Sigma). Cell viability was evaluated after a 16 h treatment period with the Cell Proliferation Reagent WST-1 (Roche), according to the manufacturer’s instructions.

### Statistical analysis

Data were expressed as means ± SEM for all mice treated similarly. Mean differences between experimental groups were assessed using the non-parametric Mann–Whitney *U*-test. Statistical analysis for the *in vitro* experiment was performed using the unpaired Student’s t-test. All statistical analysis were achieved with the GraphPad Prism5 software. Calculated *p* values are integrated on histograms and graphs.

## Electronic supplementary material

Supplementary Info
