# Supplementary material for: RIPK1 protects hepatocytes from death in Fas-induced hepatitis
Source: Sci Rep. 2017 Aug 23;7:9205. doi: 10.1038/s41598-017-09789-8 (PMC5569041; doi:10.1038/s41598-017-09789-8)
Supplement: Supplementary file 1 — Supplementary Info [file 41598_2017_9789_MOESM1_ESM.pdf]

RIPK1 protects hepatocytes from death in Fas-induced hepatitis

Aveline Filliol[1,2,3], Muhammad Farooq[1,2,3], Claire Piquet-Pellorce[1,2,3], Valentine Genet[1,2,3], Marie-Thérèse Dimanche-Boitrel[1,2,3], Peter Vandenabeele[4,5], Mathieu JM Bertrand[4,5] , Michel Samson[1,2,3,*,#] and Jacques Le Seyec[1,2,3,*]

[1]Institut National de la Santé et de la Recherche Médicale (Inserm), U.1085, Institut de Recherche en Santé, Environnement et Travail (IRSET), F-35043 Rennes, France.
[2]Université de Rennes 1, F-35043 Rennes, France.
[3]Structure Fédérative BioSit UMS 3480 CNRS-US18 Inserm, F-35043 Rennes, France.
[4]Inflammation Research Center, VIB, Technologiepark 927, Zwijnaarde-Ghent, 9052, Belgium.
[5]Department of Biomedical Molecular Biology, Ghent University, Technologiepark 927, Zwijnaarde-Ghent, 9052, Belgium.

[*] Co-senior authors
[#] Correspondence to michel.samson@univ-rennes1.fr

Figure S1

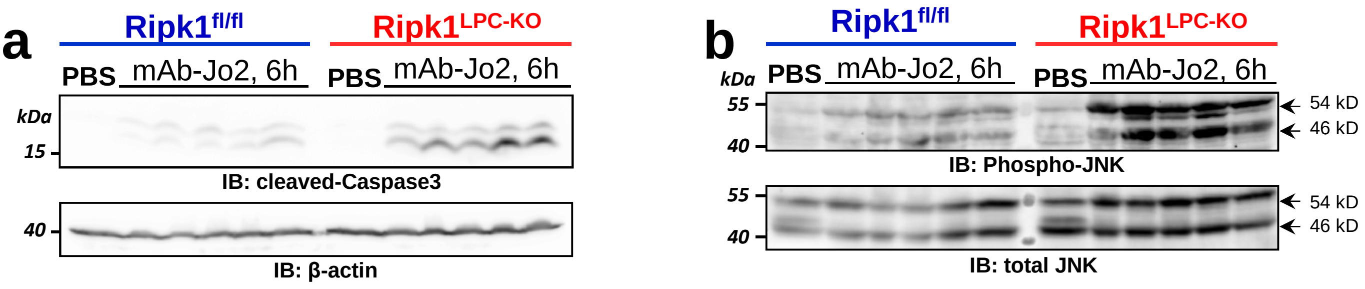

RIPK1 deficiency exacerbates the apoptotic response in Fas-induced hepatitis.

Western-blot analysis of cleaved caspase-3 and β-actin (a) or of phosphorylated-JNK and total JNK (b) in protein extracts issued from the livers of $Ripk1^{fl/fl}$ and $Ripk1^{LPC\text{-}KO}$ mice, collected 6 h after mAb-Jo2 injection. Studied proteins are respectively indicated at the bottom of the gel.

Figure S2

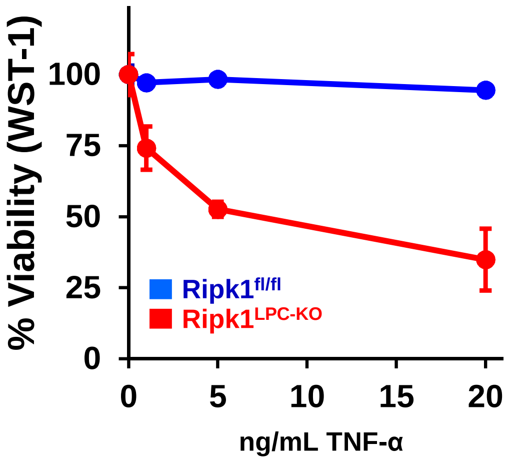

Effect of TNF-α stimulation on normal or RIPK1-deficient primary hepatocytes.

Primary cultures of hepatocytes issued from Ripk1$^{fl/fl}$ or Ripk1$^{LPC\text{-}KO}$ mice were seeded in presence of ETA. After 4 h, cell cultures were washed 2 times with PBS before being subjected during 16 h to TNF-α concentrations ranging from 1 to 20 ng/mL, in absence of ETA. Cell death were analyzed by WST-1 based assay and data are expressed in percentage of signal obtained in basal survival conditions without death factor. Error bars corresponds to SEM estimated from three independent experiments.
